# Supplementary figures and images for: The antibacterial effect of human adipose-derived stem cells on LL-37-resistant bacteria
Source: PLoS One. 2025 Oct 17;20(10):e0333647. doi: 10.1371/journal.pone.0333647 (PMC12533887; doi:10.1371/journal.pone.0333647)

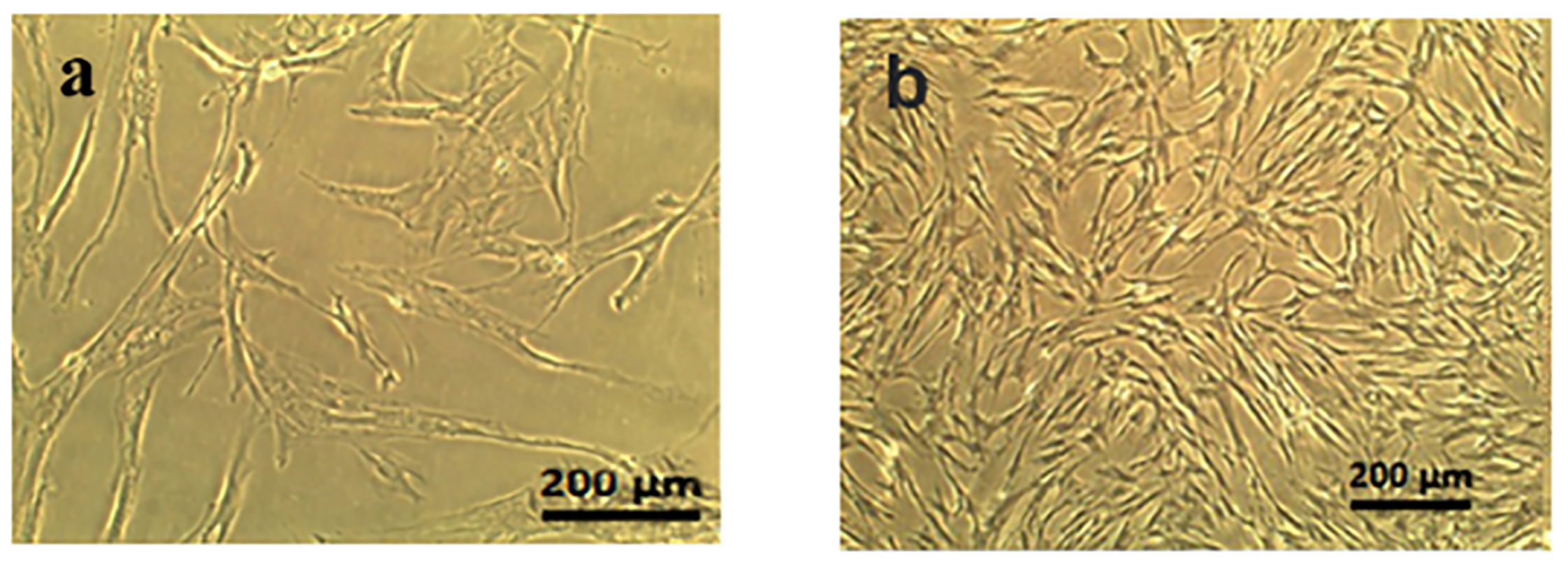

Supplement: S1 Fig — a) Proliferation on days 2–3 χ 20, b) Cells with fibroblast-like shape and 70–80% confluency χ10. (TIF) [file pone.0333647.s001.tif]

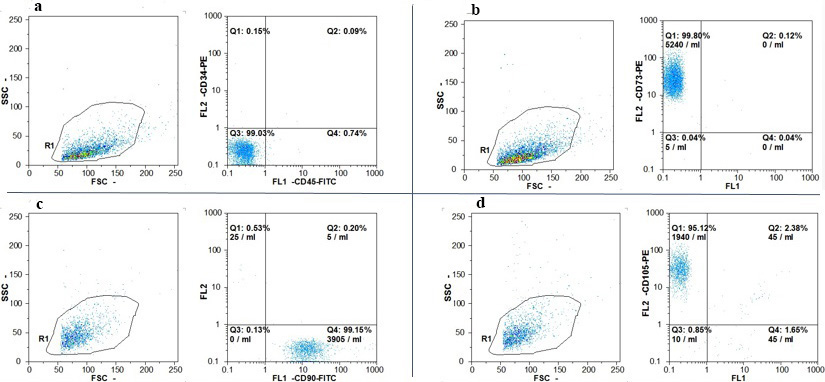

Supplement: S2 Fig — Scatter plots showed the ADSCs were positive for CD34-CD45 (a), CD73 (b) and were negative for CD90 (c) and CD105 (d). (TIF) [file pone.0333647.s002.tif]

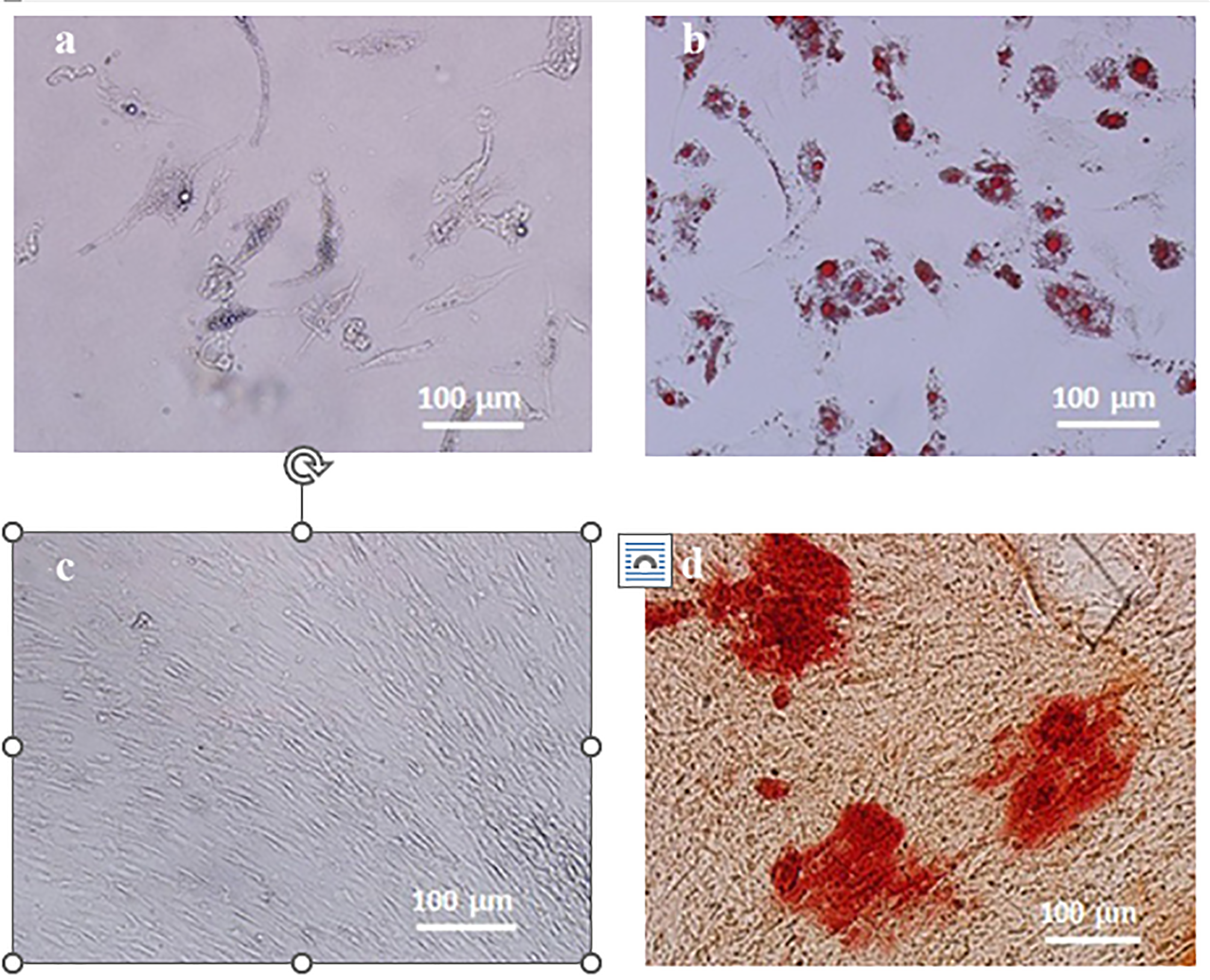

Supplement: S3 Fig — (a) Differentiated cells without staining. (b) Differentiated cells stained by Oil Red O. (c) Differentiated cells without staining. (d) Differentiated cells stained by Alizarin Red. (TIF) [file pone.0333647.s003.tif]
