# Supplementary material for: The antibacterial effect of human adipose-derived stem cells on LL-37-resistant bacteria
Source: PLoS One. 2025 Oct 17;20(10):e0333647. doi: 10.1371/journal.pone.0333647 (PMC12533887; doi:10.1371/journal.pone.0333647)
Supplement: S2 Text — Experimental details and acquisition parameters. (DOCX) [file pone.0333647.s007.docx]

Metadata for CD Markers:

CD73 conjugated with PE dye (Antibody produced by EXBIO Praha, a.s.) (FL2)

Instrument: Partec PAS flow cytometer

Software: Partec FloMax, Version 2.0.0.1)

**Acquisition settings**:

Speed: 17.3

Gains: FSC = 215, SSC = 222, FL2 (CD73-PE) = 296

Scale: FSC and SSC linear, FL2 logarithmic (log4)

Threshold and Compensation: Compensation ~999.9 (no or minimal compensation), LogBias ON

**Data collected**:

FL2-CD73-PE fluorescence intensity histogram

Scatter plots: SSC vs. FL2-CD73-PE

Cell counts and percentages in gating regions:

RN1: 8 cells (0.16%)

RN2: 4996 cells (99.9%)

Q1: 4990 cells (99.78%)

Q2: 6 cells (0.12%)

Q3: 2 cells (0.04%)

Q4: 2 cell (0.04%)

R1: 4904 cells (98.06%)
