# Supplementary material for: The antibacterial effect of human adipose-derived stem cells on LL-37-resistant bacteria
Source: PLoS One. 2025 Oct 17;20(10):e0333647. doi: 10.1371/journal.pone.0333647 (PMC12533887; doi:10.1371/journal.pone.0333647)
Supplement: S5 Text — Experimental details and acquisition parameters. (DOCX) [file pone.0333647.s013.docx]

Metadata for CD Markers:

CD45 conjugated with FITC dye (Antibody produced by EXBIO Praha, a.s.) (FL1)

CD73 conjugated with PE dye (Antibody produced by EXBIO Praha, a.s.) (FL2)

Instrument: Partec PAS flow cytometer

Software: Partec FloMax, Version 2.0.0.1)

**Acquisition settings**:

Speed: 23

Gains: FSC = 219, SSC = 222, FL1 (CD45-FITC) = 224, FL2 (CD73-PE) = 296

Scale: FSC and SSC linear, FL2 logarithmic (log4)

Threshold and Compensation: Compensation ~999.9 (no or minimal compensation), LogBias ON

**Data collected**:

FL1-CD45-FITC and FL2-CD73-PE fluorescence intensity histogram

Scatter plots: SSC vs. FL1-CD45-FITC and SSC vs. FL2-CD73-PE

Cell counts and percentages in gating regions:

RN1: 52 cells (1.04%)

RN2: 4704 cells (94.06%)

Q1: 4656 cells (93.1%)

Q2: 48 cells (0.96%)

Q3: 295 cells (5.9%)

Q4: 0 cell (0.02%)

R1: 4548 cells (90.94%)
