# Supplementary material for: The antibacterial effect of human adipose-derived stem cells on LL-37-resistant bacteria
Source: PLoS One. 2025 Oct 17;20(10):e0333647. doi: 10.1371/journal.pone.0333647 (PMC12533887; doi:10.1371/journal.pone.0333647)
Supplement: S7 Text — Experimental details and acquisition parameters. (DOCX) [file pone.0333647.s017.docx]

Metadata for CD Markers:

CD105 conjugated with PE dye (Antibody produced by EXBIO Praha, a.s.) (FL2)

Instrument: Partec PAS flow cytometer

Software: Partec FloMax, Version 2.0.0.1

**Acquisition settings**:

Speed: 23

Gains: FSC = 219, SSC = 222, FL2 (CD105-PE) = 296

Scale: FSC and SSC linear, FL2 logarithmic (log4)

Threshold and Compensation: Compensation ~999.9 (no or minimal compensation), LogBias ON

**Data collected**:

FL2-CD105-PE fluorescence intensity histogram

Scatter plots: SSC vs. FL2-CD105-PE

Cell counts and percentages in gating regions:

RN1: 66 cells (4.03%)

RN2: 1597 cells (97.44%)

Q1: 1558 cells (95.06%)

Q2: 39 cells (2.38%)

Q3: 14 cells (0.85%)

Q4: 27 cell (1.65%)

R1: 1553 cells (94.75%)
