# Supplementary material for: The antibacterial effect of human adipose-derived stem cells on LL-37-resistant bacteria
Source: PLoS One. 2025 Oct 17;20(10):e0333647. doi: 10.1371/journal.pone.0333647 (PMC12533887; doi:10.1371/journal.pone.0333647)
Supplement: S9 Text — Experimental details and acquisition parameters. (DOCX) [file pone.0333647.s021.docx]

Metadata for CD Markers:

CD73 conjugated with PE dye (Antibody produced by EXBIO Praha, a.s.) (FL2)

Instrument: Partec PAS flow cytometer

Software: Partec FloMax, Version 2.0.0.1)

**Acquisition settings**:

Speed: 16

Gains: FSC = 215, SSC = 222, FL1 (CD90-FITC) = 204, FL2 (CD105-PE) = 296

Scale: FSC and SSC linear, FL2 logarithmic (log4)

Threshold and Compensation: Compensation ~999.9 (no or minimal compensation), LogBias ON

**Data collected**:

FL2-CD73-PE fluorescence intensity histogram

Scatter plots: SSC vs. FL2-CD73-PE

Cell counts and percentages in gating regions:

RN1: 43 cells (1.87%)

RN2: 2244 cells (97.57%)

Q1: 2264 cells (98.43%)

Q2: 0 cell (0.00%)

Q3: 11 cells (0.48%)

Q4: 24 cell (1.04%)

R1: 2248 cells (97.74%)
