# Supplementary material for: The antibacterial effect of human adipose-derived stem cells on LL-37-resistant bacteria
Source: PLoS One. 2025 Oct 17;20(10):e0333647. doi: 10.1371/journal.pone.0333647 (PMC12533887; doi:10.1371/journal.pone.0333647)
Supplement: S14 Text — Experimental details and acquisition parameters. (DOCX) [file pone.0333647.s031.docx]

Metadata for CD Markers:

CD90 conjugated with FITC dye (Antibody produced by EXBIO Praha, a.s. )(FL1)

Instrument: Partec PAS flow cytometer

Software: Partec FloMax, Version 2.0.0.1)

**Acquisition settings**:

Speed: 31.2

Gains: FSC = 21, SSC = 222, FL1 (CD90-FITC) = 224

Scale: FSC and SSC linear, FL1 and FL2 logarithmic (log4)

Threshold and Compensation: Compensation ~999.9 (no or minimal compensation), LogBias ON

**Data collected**:

FL1-CD90-FITC fluorescence intensity histogram

Scatter plots: SSC vs. FL1-CD90-FITC

Cell counts and percentages in gating regions:

RN1: 680 cells (96.59%)

RN2: 6 cells (0.85%)

Q1: 4 cells (0.57%)

Q2: 2 cells (0.28%)

Q3:19 cells (2.70%)

Q4: 678 cells (96.31%)

R1: 690 cells (98.01%)
