# Supplementary material for: The antibacterial effect of human adipose-derived stem cells on LL-37-resistant bacteria
Source: PLoS One. 2025 Oct 17;20(10):e0333647. doi: 10.1371/journal.pone.0333647 (PMC12533887; doi:10.1371/journal.pone.0333647)
Supplement: S1 File — Experimental metadata including details on sample preparation, qPCR reagents and kits, primer sequences for LL-37 and GAPDH, reaction conditions, and technical duplicate measurements. (DOCX) [file pone.0333647.s035.docx]

Metadata for Real-Time PCR Experiment

Adipose-derived stem cells (ADSCs), passages 4–9, were seeded at 2×10⁵ cells per well in 24-well plates using complete DMEM supplemented with 10% FBS. After 24 hours, the medium was refreshed with DMEM containing 300 CFU of E. coli, with or without 1.25 µg/mL heparin; untreated cells served as controls. Following a 6-hour incubation, total RNA was extracted using RNX-Plus reagent, quantified by NanoDrop, and treated with DNase I to remove genomic DNA contamination. cDNA synthesis was performed using the 2X RT PRE-MIX Kit.

Specific primers for LL-37 (forward 5′-TAACCTCTACCGCCTCCTGGACCTGGACC-3′; reverse 5′-GGACTCTGTCCTGGGTACAAGATTCCGC-3′) and GAPDH (forward 5′-CAAGATCATCACCAATGCCT-3′; reverse 5′-CCCATCACGCCACAGTTTCC-3′) were designed via Integrated DNA Technologies (IDT) and validated by agarose gel electrophoresis.

Quantitative PCR was carried out on a Roche LightCycler using BioFACT™ 2X SYBR Green Master Mix. Cycling conditions were 94°C for 10 minutes, followed by 40 cycles of 94°C for 20 seconds and 72°C for 20 seconds; annealing temperatures were 60°C for both hepcidin and GAPDH. All reactions were performed in duplicate. Relative gene expression levels were calculated using the 2^(-ΔΔCt) method normalized to GAPDH and control samples.
