# Supplementary material for: The antibacterial effect of human adipose-derived stem cells on LL-37-resistant bacteria
Source: PLoS One. 2025 Oct 17;20(10):e0333647. doi: 10.1371/journal.pone.0333647 (PMC12533887; doi:10.1371/journal.pone.0333647)
Supplement: S2 File — Experimental metadata including bacterial strains used for incubation and replicates. (DOCX) [file pone.0333647.s037.docx]

Metadata for LL-37 levels under various bacterial conditions and stimulation states

This dataset contains raw experimental LL-37 concentration values (ng/ml) measured from four individual stem cell samples, each exposed to different conditions: medium only (used as a baseline control), no bacteria, *Pseudomonas aeruginosa, Proteus mirabilis*, and MRSA. Measurements were conducted under both unstimulated and IFN-γ stimulated states. The data report individual experimental values without aggregation, enabling detailed analysis of LL-37 production under different bacterial exposures and stimulation conditions.
