# Supplementary material for: The antibacterial effect of human adipose-derived stem cells on LL-37-resistant bacteria
Source: PLoS One. 2025 Oct 17;20(10):e0333647. doi: 10.1371/journal.pone.0333647 (PMC12533887; doi:10.1371/journal.pone.0333647)
Supplement: S5 File — Experimental metadata including bacterial strain and CFU counts and replicates. (DOCX) [file pone.0333647.s043.docx]

CFU data of MRSA under *E. coli* stimulated medium

This dataset contains raw bacterial colony-forming unit (CFU) counts (expressed as CFU × 10³) of MRSA obtained from in vitro experiments evaluating the efficacy of *E. coli* stimulated medium. The CFU values represent individual experimental measurements reflecting bacterial viability after exposure to these conditions. This raw data supports the interpretation of antimicrobial activity and bacterial response in the associated study and enables independent analysis of treatment effects across replicates.
