# Supplementary material for: The antibacterial effect of human adipose-derived stem cells on LL-37-resistant bacteria
Source: PLoS One. 2025 Oct 17;20(10):e0333647. doi: 10.1371/journal.pone.0333647 (PMC12533887; doi:10.1371/journal.pone.0333647)
Supplement: Attachment — Submitted filename: PONE-D-25-35482_reviewer.pdf [file pone.0333647.s050.pdf]

PLOS ONE

The antibacterial effect of human adipose-derived stem cells on LL-37-resistant bacteria

--Manuscript Draft--

**Manuscript Number:**	PONE-D-25-35482
**Article Type:**	Research Article
**Full Title:**	The antibacterial effect of human adipose-derived stem cells on LL-37-resistant bacteria
**Short Title:**	Human adipose-derived stem cells and LL-37-resistant bacteria
**Corresponding Author:**	Malek Moien Ansar, PhD<br>Guilan University of Medical Sciences<br>Rasht, IRAN, ISLAMIC REPUBLIC OF
**Keywords:**	stem cells, LL-37 antibacterial peptide, Pseudomonas aeruginosa, Proteus mirabilis, Methicillin-resistant Staphylococcus aureus
**Abstract:**	InThe antibacterial effects of human adipose-derived stem cells (hADSCs) on LL-37-resistant bacteria are not fully understood. We aimed to investigate the antibacterial efficacy of hADSCs against Pseudomonas aeruginosa, Proteus mirabilis, and methicillin-resistant Staphylococcus aureus (MRSA), focusing on the antimicrobial peptide LL-37. hADSCs were isolated from human adipose tissue, characterized by flow cytometry and differentiation assays, and stimulated with interferon-gamma (IFN-γ; 100 ng/mL) or Escherichia coli (300 CFU). LL-37 gene expression was measured by qPCR after 6 hours of bacterial stimulation. LL-37 peptide levels were quantified by ELISA in conditioned media from unstimulated, IFN-γ stimulated cells, both before and after incubation with pathogens (300 CFU). Antibacterial activity was assessed by colony counting incubation following incubation of conditioned media with bacteria. Conditioned media from both unstimulated and stimulated hADSCs significantly inhibited growth of all three pathogens ($P<0.05$), with highest efficacy against P. aeruginosa (86.4% inhibition), followed by MRSA (74%) and P. mirabilis (63%). LL-37 gene expression increased after bacterial stimulation, and also LL-37 concentrations increased in conditioned media but significantly decreased after bacterial exposure ($P<0.05$). Despite this reduction, antibacterial activity persisted. hADSC-conditioned media exert potent antibacterial effects against LL-37-resistant pathogens, even when LL-37 levels are reduced after bacterial exposure. These findings support the therapeutic potential of hADSC secretomes, particularly for infections caused by bacteria capable of reducing LL-37 levels.
**Order of Authors:**	Parisa Afzal Haghjoo
	Ali Mojtahedi
	Malek Moien Ansar, PhD
	Malek Masoud Ansar
	Safieh Danesh Mobarhan
**Additional Information:**	
**Question**	**Response**
**Financial Disclosure**<br><br>Enter a financial disclosure statement that describes the sources of funding for the work included in this submission. Review the submission guidelines for detailed requirements. View published research articles from *PLOS ONE* for specific examples.	Yes

Powered by Editorial Manager® and ProduXion Manager® from Aries Systems Corporation

This statement is required for submission and **will appear in the published article** if the submission is accepted. Please make sure it is accurate.<br><br>**Funded studies**<br>Enter a statement with the following details:<br>• Initials of the authors who received each award<br>• Grant numbers awarded to each author<br>• The full name of each funder<br>• URL of each funder website<br>• Did the sponsors or funders play any role in the study design, data collection and analysis, decision to publish, or preparation of the manuscript?<br><br>Did you receive funding for this work?	
Please add funding details.<br>as follow-up to "**Financial Disclosure**<br><br>Enter a financial disclosure statement that describes the sources of funding for the work included in this submission. Review the submission guidelines for detailed requirements. View published research articles from *PLOS ONE* for specific examples.<br><br>This statement is required for submission and **will appear in the published article** if the submission is accepted. Please make sure it is accurate.<br><br>**Funded studies**<br>Enter a statement with the following details:<br>• Initials of the authors who received each award<br>• Grant numbers awarded to each author<br>• The full name of each funder<br>• URL of each funder website<br>• Did the sponsors or funders play any role in the study design, data collection and analysis, decision to publish, or preparation of the manuscript?	Dr Malek Moien Ansar received the award.<br>Due to ongoing sanctions and war affecting our country, our research funding is extremely limited. We have no alternative funding sources available to cover publication fees and therefore request full fee assistance through the PLOS Publication Fee Assistance program<br>Grant No:96022308<br>The sponsors did not play a role in the study design, data collection and analysis, decision to publish, or preparation of the manuscript.

Powered by Editorial Manager® and ProduXion Manager® from Aries Systems Corporation

Did you receive funding for this work?"	
Please select the country of your main research funder (please select carefully as in some cases this is used in fee calculation).<br>as follow-up to "**Financial Disclosure**<br><br>Enter a financial disclosure statement that describes the sources of funding for the work included in this submission. Review the submission guidelines for detailed requirements. View published research articles from *PLOS ONE* for specific examples.<br><br>This statement is required for submission and **will appear in the published article** if the submission is accepted. Please make sure it is accurate.<br><br>**Funded studies**<br>Enter a statement with the following details:<br>• Initials of the authors who received each award<br>• Grant numbers awarded to each author<br>• The full name of each funder<br>• URL of each funder website<br>• Did the sponsors or funders play any role in the study design, data collection and analysis, decision to publish, or preparation of the manuscript?<br><br>Did you receive funding for this work?"	IRAN, ISLAMIC REPUBLIC OF - IR
**Competing Interests**<br><br>Use the instructions below to enter a competing interest statement for this submission. On behalf of all authors, disclose any competing interests that could be perceived to bias this work—acknowledging all financial support and any other relevant financial or non-financial competing interests.<br><br>This statement is required for submission	The authors have declared that no competing interests exist.

Powered by Editorial Manager® and ProduXion Manager® from Aries Systems Corporation

and **will appear in the published article** if the submission is accepted. Please make sure it is accurate and that any funding sources listed in your Funding Information later in the submission form are also declared in your Financial Disclosure statement.<br><br>View published research articles from *PLOS ONE* for specific examples.<br><br>**NO authors have competing interests**<br><br>Enter: *The authors have declared that no competing interests exist.*<br><br>**Authors with competing interests**<br><br>Enter competing interest details beginning with this statement:<br><br>*I have read the journal's policy and the authors of this manuscript have the following competing interests: [insert competing interests here]*<br><br>* typeset	
**Ethics Statement**<br><br>Enter an ethics statement for this submission. This statement is required if the study involved:<br><br>• Human participants<br>• Human specimens or tissue<br>• Vertebrate animals or cephalopods<br>• Vertebrate embryos or tissues<br>• Field research<br><br>Write "N/A" if the submission does not require an ethics statement.<br><br>General guidance is provided below. Consult the submission guidelines for detailed instructions. **Make sure that all information entered here is included in the Methods section of the manuscript.**	This study was approved by the Guilan University Ethics Committee (Ethics code: IR.GUMS.REC.1396.62, 2018) and conducted in accordance with the Declaration of Helsinki and institutional ethical guidelines. Experiments were conducted after obtaining informed consent from all participants, and all procedures were performed in accordance with relevant guidelines and regulations

**Format for specific study types**<br><br>**Human Subject Research (involving human participants and/or tissue)**<br>• Give the name of the institutional review board or ethics committee that approved the study<br>• Include the approval number and/or a statement indicating approval of this research<br>• Indicate the form of consent obtained (written/oral) or the reason that consent was not obtained (e.g. the data were analyzed anonymously)<br><br>**Animal Research (involving vertebrate animals, embryos or tissues)**<br>• Provide the name of the Institutional Animal Care and Use Committee (IACUC) or other relevant ethics board that reviewed the study protocol, and indicate whether they approved this research or granted a formal waiver of ethical approval<br>• Include an approval number if one was obtained<br>• If the study involved *non-human primates*, add *additional details* about animal welfare and steps taken to ameliorate suffering<br>• If anesthesia, euthanasia, or any kind of animal sacrifice is part of the study, include briefly which substances and/or methods were applied<br><br>**Field Research**<br><br>Include the following details if this study involves the collection of plant, animal, or other materials from a natural setting:<br>• Field permit number<br>• Name of the institution or relevant body that granted permission	
**Data Availability**<br><br>Authors are required to make all data underlying the findings described fully available, without restriction, and from the time of publication. PLOS allows rare exceptions to address legal and ethical concerns. See the PLOS Data Policy and FAQ for detailed information.	Yes - all data are fully available without restriction

A Data Availability Statement describing where the data can be found is required at submission. Your answers to this question constitute the Data Availability Statement and **will be published in the article**, if accepted.<br><br>**Important:** Stating 'data available on request from the author' is not sufficient. If your data are only available upon request, select 'No' for the first question and explain your exceptional situation in the text box.<br><br>Do the authors confirm that all data underlying the findings described in their manuscript are fully available without restriction?	
**Describe where the data may be found in full sentences. If you are copying our sample text, replace any instances of XXX with the appropriate details.**<br><br>• If the data are **held or will be held in a public repository**, include URLs, accession numbers or DOIs. If this information will only be available after acceptance, indicate this by ticking the box below. For example: *All XXX files are available from the XXX database (accession number(s) XXX, XXX.*).<br>• If the data are all contained **within the manuscript and/or Supporting Information files**, enter the following: *All relevant data are within the manuscript and its Supporting Information files.*<br>• If neither of these applies but you are able to provide **details of access elsewhere**, with or without limitations, please do so. For example:<br><br>*Data cannot be shared publicly because of [XXX]. Data are available from the XXX Institutional Data Access / Ethics Committee (contact via XXX) for researchers who meet the criteria for access to confidential data.*<br><br>*The data underlying the results presented in the study are available from (include the name of the third party*	All relevant data are within the manuscript and its Supporting Information files.

and contact information or URL).<br>• This text is appropriate if the data are owned by a third party and authors do not have permission to share the data.<br><br>* typeset	
Additional data availability information:	

Powered by Editorial Manager® and ProduXion Manager® from Aries Systems Corporation

Manuscript
Click here to access/download;Manuscript;manuscript with qPCR Plos one 2.docx

Title

The antibacterial effect of human adipose-derived stem cells on LL-37-resistant bacteria

Running title

Human adipose-derived stem cells and LL-37-resistant bacteria

Parisa Afzal Haghjoo [1, 2], Ali Mojtahedi[1], Malek Moien Ansar[3, 4 *], Malek Masoud Ansar[5], Safieh Danesh Mobarhan[5]

[1.] *Department of Microbiology, School of Medicine, Guilan Univercity of Medical Sciences, Guilan, Rasht, Iran.*

[2] *Cellular and Molecular Research Center, Guilan University of Medical Sciences, Guilan, Rasht, Iran*

[3] *Neuroscience Research Center, Trauma Institute, Guilan University of Medical Sciences, Guilan, Rasht, Iran*

[4] *Department of Biochemistry, School of Medicine, Guilan University of Medical Sciences, Guilan, Rasht, Iran*

[5] *Department of Anatomy, School of Medicine, Guilan University of Medical Sciences, Guilan, Rasht, Iran.*

Corresponding author

Email: ansar_moien@yahoo.com (MMA)

Tel: +98136690884 Fax: +981316690036

Abstract

The antibacterial effects of human adipose-derived stem cells (hADSCs) on LL-37-resistant bacteria are not fully understood. We aimed to investigate the antibacterial efficacy of hADSCs against *Pseudomonas aeruginosa, Proteus mirabilis, and methicillin-resistant Staphylococcus aureus* (MRSA), focusing on the antimicrobial peptide LL-37. hADSCs were isolated from human adipose tissue, characterized by flow cytometry and differentiation assays, and stimulated with interferon-gamma (IFN-γ; 100 ng/mL) or *Escherichia coli* (300 CFU). L[illegible] gene expression was measured by qPCR after 6 hours of bacterial stimulation. LL-37 peptide levels were quantified by ELISA in conditioned media from unstimulated, IFN-γ stimulated cells, both before and after incubation with pathogens (300 CFU). Antibacterial activity was assessed by colony counting incubation following incubation of conditioned media with bacteria. Conditioned media from both unstimulated and stimulated hADSCs significantly inhibited growth of all three pathogens ($P<0.05$), with highest efficacy against *P. aeruginosa* (86.4% inhibition), followed by MRSA (74%) and *P. mirabilis* (63%). LL-37 gene expression increased after bacterial stimulation, and also LL-37 concentrations increased in conditioned media but significantly decreased after bacterial exposure ($P<0.05$). Despite this reduction, antibacterial activity persisted. hADSC-conditioned media exert potent antibacterial effects against LL-37-resistant pathogens, even when LL-37 levels are reduced after bacterial exposure. These findings support the therapeutic potential of hADSC secretomes, particularly for infections caused by bacteria capable of reducing LL-37 levels.

Introduction

Infectious diseases caused by bacteria, viruses, or parasites are responsible for many deaths worldwide. Due to the global rise in drug-resistant infections, there has been increasing interest in developing new treatments and approaches [1, 2]. Recently, the potential of stem cells to treat infections has gained attention [3]. One probable mechanism underlying the antimicrobial effects of mesenchymal stromal/stem cells (MSCs) is the secretion of antimicrobial peptides (AMPs) [4, 5]. AMPs exhibit selective activity against a wide range of organisms, including bacteria, yeasts, fungi, viruses, and even cancer cells [4]. The best-known human antimicrobial peptide is LL-37- the only member of human Cathelicidin family- which is found in various tissues and body fluids and can be secreted by different cell types [6] including MSCs [7-9]. The antimicrobial effects of LL-37 are mediated by direct interactions with microbial cell membrane, leading to disruption of membrane integrity and the immunomodulatory effects on inflammatory cells [10]. Despite recent research, studies on the antibacterial effects of human adipose-derived stem cells (hADSCs) remain limited [11]. It has been shown that different stem cell types may have distinct antimicrobial effects depending on both the cell type and the pathogen [7, 9]. Furthermore, many studies have reported that LL-37 is the main mediator of antibacterial action of MSCs [7)] but some bacteria secrete proteases that may degrade LL-37.

In this study, we investigated the antibacterial effect of hADSCs against three clinically relevant strains: Methicillin-resistant *Staphylococcus aureus* (MRSA), *Proteus mirabilis* and *Pseudomonas aeruginosa*. MRSA continues to be a major multidrug-resistant pathogen causing serious infections worldwide, with rising concerns over antibiotic resistance [12] *P. mirabilis* resists the antimicrobial peptide LL-37 primarily through secretion of proteases such as ZapA that degrade this peptide [13]. *P. aeruginosa* is a notorious opportunistic pathogen, largely due to its biofilm

formation and intrinsic resistance mechanisms that complicate treatment [14]. Recent studies have shown that hADSCs secrete LL-37 and other antimicrobial factors, contributing to their broad antibacterial potential [15]

We focused on assessing the antibacterial effects of hADSCs on LL-37-resistant bacteria, including *P. mirabilis* and MRSA [13, 16], as well as *P. aeruginosa* under *in vitro* conditions.

Materials and methods

Ethics and sample collection

This study was approved by the Guilan University Ethics Committee (Ethics code: IR.GUMS.REC.1396.62) and conducted in accordance with the Declaration of Helsinki and institutional ethical guidelines. Participants were recruited between 30 August 2017 and 10 January 2018. Written informed consent was obtained and all procedures adhered to relevant guidelines and regulations.

Adipose tissue samples were collected from four unrelated healthy subjects undergoing liposuction or adipose tissue dissection for weight loss. Active standard bacterial strains used for antibacterial assays were procured from the Pasteur Institute and the National Center for Genetic and Biological Resources, Iran.

Isolation and Culture of hADSCs

Mesenchymal stem cells were isolated from adipose tissue samples using the enzymatic digestion method described by Bunnell et al. (17). Briefly, adipose tissue was washed with phosphate-buffered saline (PBS), mechanically minced, and incubated in 0.25% collagenase type I solution for 40 minutes at 37°C. The cell suspension was centrifuged at 1200 rpm for 10 minutes to collect the cell pellet. Collagenase activity was neutralized by adding Dulbecco's Modified Eagle Medium

(DMEM) supplemented with 10% fetal bovine serum (FBS) and penicillin-streptomycin. The pellet was resuspended in the same medium and seeded into tissue culture flasks, designated as passage 0, and incubated at 37°C in a humidified atmosphere containing 5% CO_2. The culture medium was refreshed every three days.

When cells reached approximately 70–80% confluency, they were rinsed twice with PBS, detached using trypsin-EDTA, and incubated at 37°C for 2 minutes. Trypsin activity was neutralized by adding culture medium. After centrifugation at 1200 rpm for 5 minutes, the cell pellet was resuspended and split into three flasks for further expansion.

Cryopreservation and Experimental Design

Isolated stem cells were cryopreserved following established protocols to maintain viability and functionality (18). The main experiments were conducted after cryopreservation to ensure consistent cell viability and functionality, allowing for standardized timing and reproducibility of assays. All experiments were performed in triplicate.

Flow cytometry analysis

Cells at passage 2 were used for flow cytometry analysis (8). Approximately 30,000 cells in 50 µL PBS were incubated with 2 µL of phycoerythrin (PE)- or fluorescein isothiocyanate (FITC)-conjugated antibodies against CD90, CD105, CD73, CD34 and CD45. After washing with PBS, the pellet was resuspended in 500µl PBS and transferred to flow cytometry tubes. Data were analyzed using FloMax software.

Adipogenic and osteogenic differentiation assay

For differentiation assays, 10,000 cells per well (adipogenic) and 6000 cells per well (osteogenic) were seeded in a 24- well plates. After 24 hours, the culture medium was replaced with 1 ml of

differentiation medium. The adipogenic medium consisted of DMEM high glucose supplemented with 10% FBS, 10^{-7} M dexamethasone, 0.5 mM IBMX, 66 nM insulin, and 0.2 mM indomethacin, The osteogenic medium consisted DMEM high glucose supplemented with 10% FBS, 10^{-7} M dexamethasone, and 50 µg/ml Ascorbic acid bi- phosphate. The differentiation medium was changed every three days. After 21 days, cells were fixed with 10% formalin for 1 hour at room temperature and stained with Oil Red O to detect lipid droplets. On day 15, cells were fixed and stained with Alizarin Red to detect calcium deposits**.** Cells from passage 3 onwards were used for these assays.

Preparation of Supernatant and Conditioned medium from hADSC

Cells from passages 4 to 9 were used for experiments (8). Medium from non-stimulated adipose-derived stem cells was prepared by seeding 200,000 cells per well in 24-well plate and culturing for 24 hours. The medium was then replaced with antibiotic-free medium. After an additional 24 hours, the medium was collected, centrifuged, and the clear supernatant stored in a -20° C (19.20). For conditioned medium from stem cells stimulated by *Escherichia coli (E. coli)*: bacteria were cultured overnight in LB broth at 37° C with gentle agitation and then plated on LB agar. After 24 hours, fresh bacterial colonies were dissolved in 2 ml of PBS and incubated for 3 hours. A bacterial suspension at 0.5 McFarland standard was prepared, diluted to 300 CFU/30 µL with PBS, and added to 470 µl of DMEM containing 10% FBS in wells containing 200,000 stem cells at 37° C. After 6 hours, the medium was collected, centrifuged, filtered, and stored at -20° C. For medium from stem cells stimulated with interferon-gamma (IFN-γ), cells were incubated with 500 µL DMEM containing 10% FBS and IFN-γ at concentration of 100ng/µL (or 2000 U/µL) (6).

Antimicrobial activity

Four bacterial standard strains were tested: *E. coli* ATCC 25922, *P. aeruginosa* ATCC 27853, *P. mirabilis* ATCC 7002 and MRSA ATCC 33591. Bacteria were cultured overnight in BHI broth at 37 °C with gentle agitation and then plated on blood agar. After 24 hours, fresh colonies were suspended in 2 ml of LB broth and adjusted to 0.5 McFarland standard. The suspension was diluted to 300 CFU/30 µL with LB broth. In a 24-well plate, 570µL of each collected medium were incubated with 30µl of bacterial suspension for 6 hours at 37° C. Bacterial counts were determined by serial dilution, and inhibition rates were compared to control. Gentamicin was used as the positive control, with serial two-fold dilutions (final concentration 100 to 0.39 µg/ml). Each well was inoculated with 300 CFU of bacteria and incubated for 6 hours. Gentamicin-treated samples were plated on appropriate media (LB agar for *E. coli and P. aeruginosa*, EMB agar for *P. mirabilis*, and blood agar for MRSA). Colony counts were performed after 24 hours. The effective gentamicin concentrations for *P. aeruginosa*, *P. mirabilis,* and MRSA were 6.25, 3.12, and 12.5 µg/mL, respectively.

Quantitative Real-Time PCR (qPCR)

ADSCs (passages 4–9) were seeded at 2×10^5 cells per well in 24-well plates with complete DMEM containing 10% FBS. After 24 hours, the medium was replaced with fresh DMEM supplemented with 300 CFU E. coli, [illegible] or without 1.25 µg/mL heparin; untreated cells served as controls. Following 6 hours of incubation, total RNA was extracted using RNX-Plus, quantified by NanoDrop, and treated with DNase I to eliminate genomic DNA contamination. cDNA synthesis was performed using the 2X RT PRE-MIX Kit. Specific primers for LL-37 — forward 5′-TAACCTCTACCGCCTCCTGGACCTGGACC-3′ and reverse 5′-GGACTCTGTCCTGGGTACAAGATTCCGC-3′ — and GAPDH — forward 5′-CAAGATCATCACCAATGCCT-3′ and reverse 5′-CCCATCACGCCACAGTTTCC-3′ — were

designed via IDT and validated by agarose gel electrophoresis. Quantitative PCR was carried out on a Roche LightCycler using BioFACT™ 2X SYBR Green Master Mix under cycling conditions of 94°C for 10 minutes, followed by 40 cycles of 94°C for 20 seconds and 72°C for 20 seconds; annealing temperatures were set at 60°C for hepcidin and GAPDH. All reactions were performed in duplicate, and relative gene expression levels were determined using the 2^(-ΔΔCt) method normalized to GAPDH and control samples.

LL-37 Measurements

Levels of human LL-37 in collected media were measured using an ELISA kit (Zellbio, Germany, Cat No: ZB-12197S-H9648), following the manufacturer's instructions.

Statistical analysis

Data were analyzed using SPSS 22 software (IBM, USA). All experiments were performed in triplicate using ADSCs derived from four unrelated healthy donors. Results are presented as mean± SEM. The Mann-Whitney U test was used to compare two independent groups. $P < 0.05$ was considered statistically significant.

Results

Morphological Characteristics of hADSCs

After initial seeding, stem cells adhered to the flask surface within 2-3 days and displayed a fibroblast-like morphology S1a Fig. Approximately ten days later, these fibroblast-like cells persisted and reached a confluency of approximately 70-80% S1b Fig.

Flow Cytometry analysis of different CD Markers

hADSCs expressed high levels of MSC markers. The expression levels of CD73, CD90, and CD105 were 98.99 ±0.34%, 96.86 ±1.24%, and 97.35 ± 1.1%, respectively. The expression of

CD34 (0.19 ±0.12%) and CD45 (1.14 ± 0.28%) was low, indicating the absence of endothelial and hematopoietic markers S2 Fig.

Adipogenic and osteogenic differentiation

The cells differentiated into adipocytes and osteocytes under specific differentiation conditions. Adipogenic differentiation was confirmed by intracellular lipid droplets stained with Oil Red O S3a and S3b Fig, and osteogenic differentiation was indicated by mineralization, visualized by Alizarin Red staining S3c and S3d Fig.

LL-37 expression and secretion

LL-37 expression in hADSCs increased by 2.54± 0.759fold following 6 hours of *E. coli* stimulation compared to control, as determined by qPCR Fig 1, indicating rapid transcriptional activation. Correspondingly, LL-37 peptide concentrations in conditioned media increased after 24 hours of culture with or without IFN-γ stimulation, as determined by ELISA. However, LL-37 protein levels decreased following incubation with bacteria, likely due to bacterial protease degradation ($F (7, 47) = 8.5$, $P=0.000$). Post-hoc analysis showed significant reductions with *P. aeruginosa* and *P. mirabilis* ($P<0.05$ and $P<0.01$ for unstimulated and IFN-γ-stimulated cells, respectively), and with MRSA at $P<0.01$ for both conditions Fig 2. These results demonstrate that LL-37 gene induction precedes peptide secretion, and secreted LL-37 is subject to proteolytic reduction in the presence of pathogens

Fig 1. qPCR LL-37 gene expression. Relative hepcidin gene expression normalized to GAPDH in ADSCs and *E. coli*-stimulated. All reactions were performed in duplicate. Data are mean± SEM. ADSC: adipose-derived stem cells; *E. coli*: 300 CFU; • $P < 0.05$ compared to ADSC group.

Fig 2: LL-37 concentration. LL-37 values were shown in medium obtained from non-stimulated hADSCs and stimulated by interferon gamma. Both mediums incubated without bacteria, *P.*

aeruginosa, *P. mirabilis* and MRSA separately. 570 µl of each collected medium was incubated with 300 CFU of three bacteria (in 30 µl of PBS) and incubated for 6 h. All media were collected and the levels of LL-37 were measured. The data represent triplicate measurements using hADSCs from four healthy unrelated donors and are presented as mean± SEM. (n: 7 to 11) * P<0.05 versus no bacteria (unstimulated) # P<0.01 versus no bacteria (IFN-ɣ stimulated).). * P<0.01, CFU: Colony-Forming Unit

Antimicrobial Activity of hADSCs

Growth of *P. aeruginosa* (Fig 3a), *P. Mirabilis* (Fig 3b) and MRSA (Fig 3c) in hADSCs-conditioned medium from *E. coli*-stimulated cells was reduced compared to the controls (P<0.05). The percentage of growth inhibition was: *P. aeruginosa* (86.4%), *P. Mirabilis* (63%), and MRSA (74%). Additionally, both unstimulated and IFN-γ stimulated hADSCs showed significant inhibition of all three bacteria compared to the controls (F (3, 22) =9.88, P=0.000, F (3 ,20) = 11.31, P=0.000, F (3, 19) = 13.29, P=0.000, respectively) Fig 4a and 4b and 4c.

Fig 3: Antibacterial activity of medium derived from hADSCs stimulated by *E. coli* (ECM) on the growth of *P. aeruginosa, P. mirabilis* and MRSA. (a) 570µl of each collected medium was incubated with 300 CFU of *P. aeruginosa* (in 30µl of PBS). (b) 570 µl of each collected medium was incubated with 300 CFU of *P. mirabilis* (in 30 µl of PBS) (c) 570 µl of each collected medium was incubated with 300 CFU of MRSA (in 30 µl of PBS). All samples were incubated for 6 h, and bacterial counts in each well determined by serial dilution. The data represent triplicate measurements using hADSCs from four healthy unrelated donors and are presented as mean± SEM. (n: 7 to 11). * P<0.05, CFU: Colony-Forming Unit;

Fig 4: Antibacterial activity of medium derived from non-stimulated hADSCs (S) and stimulated by interferon gamma (ICM) on the growth of *P. aeruginosa, P. mirabilis* and *MRSA*. (a) 570 µl of each collected medium was incubated with 300 CFU of *P. aeruginos*(in 30 µl of PBS), b) 570µl of each collected medium was incubated with 300 CFU of *P. mirabilis* (in 30µl of PBS), c) 570 µl of each collected medium was incubated with 300 CFU of MRSA (in 30 µl of PBS) incubated for 6 h. Bacterial counts in each well determined by serial dilutions. The data represent triplicate measurements using hADSCs from four healthy unrelated donors and are presented as mean± SEM. (n: 7 to 11). Gentamicin was used as positive control at a concentration of 6.25µg/ml. * $P<0.05$, CFU: Colony-Forming Unit

Discussion

Our results demonstrated that the conditioned media from hADSCs significantly inhibited the growth of MRSA, *P. mirabilis, and P. aeruginosa* with the highest inhibition observed against *P. aeruginosa* (86.4%), followed by MRSA (74%) and *P. mirabilis* (63%). Despite a significant reduction in LL-37 peptide levels in conditioned media after bacterial exposure—likely due to bacterial protease activity—antibacterial activity persisted, indicating that LL-37 is not the sole mediator of this effect. Quantitative PCR analysis revealed that LL-37 gene expression in hADSCs increased approximately 2.54-fold after 6 hours of *E. coli* stimulation, confirming that LL-37 production is inducible in response to bacterial challenge.

The measurement of LL-37 gene expression by qPCR after 6 hours of *E. coli* exposure captures early transcriptional responses of hADSCs to bacterial challenge. This rapid induction precedes detectable increases in secreted peptide levels measured by ELISA after 24 hours of culture with or without IFN-γ stimulation. The observed decrease in LL-37 protein following bacterial incubation reflects proteolytic degradation by bacterial enzymes known to degrade antimicrobial peptides. Together, these complementary assays elucidate the kinetics of LL-37 production and degradation, highlighting the dynamic interplay between stem cell antimicrobial responses and bacterial evasion strategies.

This dual measurement approach strengthens the mechanistic understanding of hADSC antimicrobial activity by linking gene induction with peptide secretion and degradation dynamics. It also supports the conclusion that, despite protease-mediated LL-37 reduction, additional secreted factors contribute to the sustained antibacterial effects of hADSC-conditioned media.

Previous studies have demonstrated that the antibacterial activity of both stimulated or non-stimulated BMSCs is primarily mediated by the secretion of LL-37 [8, 9], with one study reporting increased LL-37 gene expression exclusively in stimulated cells [8]. Among eight studies reporting LL-37 secretion by MSCs [11], only one focused on hADSCs and examined LL-37 expression following vitamin D exposure [21]. Conversely, Miranda et al. showed that LL-37 gene expression was not induced in stimulated BMSCs or menstrual stem cells by lipopolysaccharide or bacterial exposure, identifying hepcidin as the major antibacterial agent instead [20].

In our study, LL-37 secretion significantly increased in both unstimulated and IFN-γ-stimulated hADSCs. Previous studies on bone marrow-derived MSCs have often emphasized LL-37 as the primary antibacterial effector; however, our findings and limited prior research on hADSCs suggest tissue-specific differences in antimicrobial profiles. For example, while synthetic LL-37

is ineffective against *P. mirabilis* due to protease-mediated degradation, hADSC-conditioned media demonstrated stronger antibacterial activity, implying synergistic effects of multiple secreted factors.

Conditioned media from unstimulated and IFN-γ-stimulated hADSCs were used for our experiments, while media from *E. coli*-stimulated cells were excluded due to bacterial protease-mediated degradation of LL-37, which interferes with accurate quantification [22]. This approach provided clearer insights into the dynamics of LL-37 expression and degradation, reinforcing the potential of hADSC secretomes as cell-free antimicrobial agents.

We demonstrated that, despite a significant reduction in LL-37 levels following exposure to *P. aeruginosa*, *P. mirabilis and* MRSA exposure, conditioned media from both stimulated and unstimulated hADSCs maintained strong antibacterial activity, highlighting the contribution of multiple antimicrobial peptides and factors beyond LL-37 [4, 8]. Given the similar effects observed across these bacterial strains, we proceeded to analyze and interpret the responses of each strain separately.

P. aeruginosa is a common nosocomial pathogen and the second most frequent cause of ventilator-associated pneumonia [23]. Its ability to form biofilms is critical for survival. LL-37 is the only cathelicidin known to possess anti-biofilm activity [24]. In our study, the antibacterial effects against P. aeruginosa persisted despite reduced LL-37 levels, suggesting that residual LL-37 or other antimicrobial factors secreted by hADSCs contribute to bacterial inhibition. Notably, P. aeruginosa showed the greatest growth inhibition among tested bacteria in hADSCs-conditioned media. This supports previous finding that P. aeruginosa is sensitive to LL-37 despite production of proteases like elastase that degrade LL-37 [25]. Our data reveal that hADSC-conditioned media retain potent activity against P. aeruginosa despite reduced LL-37 levels, supporting the hypothesis

that residual LL-37 and other secreted antimicrobial components act in concert to inhibit bacterial growth. Therefore, hADSC-conditioned media, even without stimulation, may be a potential therapeutic agent against P. aeruginosa infections.

To our knowledge, this is the first report demonstrating the antimicrobial activity of hADSCs-conditioned media against P. mirabilis and implicating LL-37 in this effect. P. mirabilis is resistant to synthetic LL-37 [12,26], likely due to ZapA, an extracellular matrix metalloprotease that degrades antimicrobial peptides such as hBD1 and LL-37 [12]. We observed a decrease in LL-37 levels in conditioned media exposed to P. mirabilis, yet the antibacterial activity of conditioned media remained stronger than that reported for synthetic LL-37 alone [12]. This suggests that (a) LL-37 is partially degraded by ZapA; (b) residual LL-37 is still effective, and (c) other antibacterial factors secreted by hADSCs contribute to the activity. The enhanced potency of ADSCs-derived LL-37 likely reflects its natural context and synergy with additional antimicrobial agents in the conditioned media.

P. mirabilis causes symptomatic urinary tract infections including cystitis and pyelonephritis, which may progress to complications such as bacteremia and potentially life-threatening urosepsis [27]. Given the antibacterial potency of hADSC-conditioned media and LL-37 secretion, these media, even without additional stimulation, may represent a promising therapeutic approach for such infections.

The antimicrobial effects of hCAP18/LL-37, LL-37 and recombinant Cathelin on MRSA ATCC 33591 have shown that this strain is resistant to the peptides hCAP18/LL-37 and LL-37 peptides, but remains sensitive to Cathelin [16]. In our study, we demonstrated that hADSCs effectively reduced MRSA (ATCC 33591), despite a decrease in LL-37 concentration. This reduction may be attributed to the ability of MRSA to secrete proteases. Previous studies have identified several

proteases secreted by MRSA including aureolysin, V8 protease, staphopain A (ScpA) and ScpB. Notably, ScpB has been reported to degrade LL-37 at concentrations higher than those observed in our study [28, 29]. To date, only two studies have investigated the interaction between MRSA and stem cells: one utilized BMSCs and focused on LL-37 expression without quantifying its concentration [30], while the other examined the effect of hADSCs secretome on MRSA without characterizing the specific secreted factors involved [15].

Given the high incidence of MRSA, accounting for up to 74% of S. aureus infections worldwide [31], the emergence of vancomycin-resistant MRSA strains, and adverse antibiotic effects, further research is warranted to hADSC antimicrobial activity against MRSA and to elucidate underlying mechanisms.

While this study focused on *in vitro* experiments to delineate fundamental antibacterial mechanisms, these findings provide a strong rationale for future *in vivo* studies to evaluate the therapeutic potential, safety, and pharmacokinetics of hADSC secretomes in relevant infection models. Additionally, further characterization of the non-LL-37 antimicrobial components in hADSC secretions is warranted to fully understand and optimize their clinical application.

Conclusion

This study demonstrates that hADSC significantly inhibit the growth of multidrug-resistant MRSA, LL-37-resistant *P. mirabilis*, and clinically relevant *P. aeruginosa*. Upon bacterial stimulation, LL-37 gene expression in hADSCs is inducible, and conditioned media from hADSC - stimulated with or without IFN-γ - show enhanced LL-37 secretion and strong antibacterial activity. Notably, despite a reduction in LL-37 concentration, the conditioned media maintain robust antibacterial effect, indicating that may residual LL-37 or possibly other secreted factors secreted by hADSCs contribute to this activity. These finding underscore the antimicrobial

potential of hADSC secretomes and support their development as promising alternative or adjunctive therapies against resistant and difficult-to-treat bacterial infections, especially those capable of reducing LL-37 levels. Future *in vivo* studies are essential to validate these results, further elucidate the underlying mechanisms, and assess the therapeutic efficacy and safety of hADSC- derived treatments.

Acknowledgment:

We dedicated our special thanks to Mr. Mostafa Haghiri (MA in English linguistics) for editing the manuscript

Declaration of Competing Interest

None of the authors have any potential conflicts of interest associated with this research.

Funding declaration

This work was supported by Guilan University of Medial Sciences .The financial support was provided for this work to cover the experimental costs, but not the publication fees.

References

1.B. A. Aderibigbe. Metal-based nanoparticles for the treatment of infectious diseases. Molecules. 2017;22(8):1370. https://doi.org/10.3390/molecules

2.World Health Organization. Antimicrobial resistance. Geneva: World Health Organization; 2023.

3.E. Lombardo, T. Van der Poll, O. DelaRosa, W. Dalemans. Mesenchymal stem cells as a therapeutic tool to treat sepsis. World J Stem Cells. 2015;7(2):368-379. https://doi.org/10.4252/wjsc.v7.i2.368

4.Y. Huan, Q. Kong, H. Mou, H. Yi. Antimicrobial peptides: classification, design, application and research progress in multiple fields. Front Microbiol. 2020; 11:582779. https://doi.org/10.3389/fmicb.2020.582779

5.G. Műzes, F. Sipos. Mesenchymal stem cell-derived secretome: A potential therapeutic option for autoimmune and immune-mediated inflammatory diseases. Cells. 2022;11:2300. https://doi.org/10.3390/cells11152300

6.U. H. Dürr, U. S. Sudheendra, A. Ramamoorthy. LL-37, the only human member of the cathelicidin family of antimicrobial peptides. Biochim Biophys Acta. 2006;1758(9):1408-1425. https://doi.org/10.1016/j.bbamem. 2006.03.030

7.F. Alcayaga-Miranda, J. Cuenca, M. Khoury. Antimicrobial activity of mesenchymal stem cells: current status and new perspectives of antimicrobial peptide-based therapies. Front Immunol. 2017;8:339. https://doi.org/10.3389/fimmu.2017.00339

8. A. Krasnodembskaya, Y. Song, X. Fang, N. Gupta, V. Serikov, J. W. Lee, M. A. Matthay. Antibacterial effect of human mesenchymal stem cells is mediated in part from secretion of the antimicrobial peptide LL-37. Stem Cells. 2010;28(12):2229-2238. https://doi.org/10.1002/stem.544

9. M. T. Sutton, D. Fletcher, S. K. Ghosh, A. Weinberg, R. van Heeckeren, S. Kaur, et al. Antimicrobial properties of mesenchymal stem cells: therapeutic potential for cystic fibrosis infection, and treatment. Stem Cells Int. 2016;2016:5303048. https://doi.org/10.1155/2016/5303048

10. J. Agier, E. Brzezińska-Błaszczyk, P. Żelechowska, M. Wiktorska, J. Pietrzak, S. Różalska. Cathelicidin LL-37 affects surface and intracellular Toll-like receptor expression in tissue mast cells. J Immunol Res. 2018;2018:7357162. https://doi.org/10.1155/2018/7357162

11. A. Castro Ramos, M. Y. Widjaja Lomanto, C.-K. Vuong, O. Ohneda, M. Fukushige. Antibacterial effects of human mesenchymal stem cells and their derivatives: a systematic review. Front Microbiol. 2024;15:1430650. https://doi.org/10.3389/fmicb.2024.1430650

12.S. Kumar, R. P. Mahato, Ch. S, S. Kumbham. Current strategies against multidrug-resistant Staphylococcus aureus and advances toward future therapy. The Microbe. 2025;6. https://doi.org/10.1016/j.microb.2025.100281

13. R. Belas, J. Manos, R. Suvanasuthi. Proteus mirabilis ZapA metalloprotease degrades a broad spectrum of substrates, including antimicrobial peptides. Infect Immun. 2004;72(9):5159-5167. https://doi.org/10.1128/IAI.72.9.5159-5167.2004

14. A. Haidar, A. Muazzam, A. Nadeem, R. Atique, H. A. Saeed, A. Naveed, J. Sharif, A. Perveen, H. R. Fatima, A. Samad. Biofilm formation and antibiotic resistance in Pseudomonas aeruginosa. The Microbe. 2024;3. https://doi.org/10.1016/j.microb.2024.100078

15. F. Shaaban, E. Salem Sokhn, C. Khalil, F. A. Saleh. Antimicrobial activity of adipose-derived mesenchymal stromal cell secretome against methicillin-resistant Staphylococcus aureus. Stem Cell Res Ther. 2025;16(1):21. https://doi.org/10.1186/s13287-025-04138-3

16. M. Zaiou, V. Nizet, R. L. Gallo. Antimicrobial and protease inhibitory functions of the human cathelicidin (hCAP18/LL-37) prosequence. J Invest Dermatol. 2003;120(5):810-816. https://doi.org/10.1046/j.1523-1747.2003.12132.x

17, Bunnell BA, Flaat M, Gagliardi C, Patel B, Ripoll C. Adipose-derived stem cells: isolation, expansion and differentiation. Methods. 2008 ;45(2):115-20. http://doi.org/10.1016/j.ymeth.2008.03.006

18. Phinney DG, Prockop DJ. Concise review: mesenchymal stem/multipotent stromal cells: the state of transdifferentiation and modes of tissue repair--current views. Stem Cells. 2007;25(11):2896-902. http://doi.org/10.1634/stemcells.2007-0637

19. V. Johnson, T. Webb, A. Norman, J. Coy, J. Kurihara, D. Regan, S. Dow. Activated mesenchymal stem cells interact with antibiotics and host innate immune responses to control chronic bacterial infections. Sci Rep. 2017;7(1):9575. https://doi.org/10.1038/s41598-017-08311-4

20.F. Alcayaga-Miranda, J. Cuenca, A. Martin, L. Contreras, F. E. Figueroa, M. Khoury. Combination therapy of menstrual derived mesenchymal stem cells and antibiotics ameliorates survival in sepsis. Stem Cell Res Ther. 2015;6:199. https://doi.org/10.1186/s13287-015-0192-0

21.H. Yagi, A. F. Chen, D. Hirsch, A. C. Rothenberg, J. Tan, P. G. Alexander, R. S. Tuan. Antimicrobial activity of mesenchymal stem cells against Staphylococcus aureus. Stem Cell Res Ther. 2020;11(1):293. https://doi.org/10.1186/s13287-020-01807-3

22.A. Schmidtchen, I. M. Frick, E. Andersson, H. Tapper, L. Björck. Proteinases of common pathogenic bacteria degrade and inactivate the antibacterial peptide LL-37. Mol Microbiol. 2002;46(1):157-168. https://doi.org/10.1046/j.1365-2958.2002.03194.x

23. S. L. Gellatly, R. E. Hancock. Pseudomonas aeruginosa: new insights into pathogenesis and host defenses. Pathog Dis. 2013;67(3):159-173. https://doi.org/10.1111/2049-632X.12033

24. D. Vandamme, B. Landuyt, W. Luyten, L. Schoofs. A comprehensive summary of LL-37, the factotum human cathelicidin peptide. Cell Immunol. 2012;280(1):22-35. https://doi.org/10.1016/j.cellimm.2012.11.009

25. A. Grönberg, L. Zettergren, M. S. Agren. Stability of the cathelicidin peptide LL-37 in a non-healing wound environment. Acta Derm Venereol. 2011;91(5):511-515. https://doi.org/10.2340/00015555-1102

26. A. A. Strömstedt, M. Pasupuleti, A. Schmidtchen, M. Malmsten. Evaluation of strategies for improving proteolytic resistance of antimicrobial peptides by using variants of EFK17, an internal segment of LL-37. Antimicrob Agents Chemother. 2009;53(2). https://doi.org/10.1128/aac.00477-08

27.J. N. Schaffer, M. M. Pearson. Proteus mirabilis and urinary tract infections. Microbiol Spectr. 2015;3(5). https://doi.org/10.1128/microbiolspec.UTI-0017-2013

28.A. Sonesson, et al. Identification of bacterial biofilm and the Staphylococcus aureus derived protease, staphopain, on the skin surface of patients with atopic dermatitis. Sci Rep. 2017;7(1):8689. https://doi.org/10.1038/s41598-017-08046-2

29. J. F. Shurko, R. S. Galega, C. Li, G. C. Lee. Evaluation of LL-37 antimicrobial peptide derivatives alone and in combination with vancomycin against S. aureus. J Antibiot (Tokyo). 2018;71(11):971-974. https://doi.org/10.1038/s41429-018-0090-7

30.L. Chow, V. Johnson, R. Impastato, J. Coy, A. Strumpf, S. Dow. Antibacterial activity of human mesenchymal stem cells mediated directly by constitutively secreted factors and indirectly by activation of innate immune effector cells. Stem Cells Transl Med. 2020;9(2):235-249. https://doi.org/10.1002/sctm.19-0092

31. A. Hassoun, P. K. Linden, B. Friedman. Incidence, prevalence, and management of MRSA bacteremia across patient populations—a review of recent developments in MRSA management and treatment. Crit Care. 2017;21(1):211. https://doi.org/10.1186/s13054-017-1801-3

Supporting Information

S1 Fig. Morphological impacts of human stem cells derived from adipose tissue. a) Proliferation on days 2-3 x 20, b) Cells with fibroblast-like shape and 70-80% confluency x10.

S2 Fig. Flow cytometry analysis of ADSCs markers expression. Scatter plots showed the ADSCs were positive for CD34-CD45 (a), CD73 (b) and were negative for CD90 (c) and CD105 (d).

S3 Fig. Adipocyte Stem cell differentiation capacity in adipogenic and osteogenic differentiation medium. (A) Differentiated cells without staining. (B) Differentiated cells stained by Oil Red O. (C) Differentiated cells without staining. (D) Differentiated cells stained by Alizarin Red.

Click here to access/download;Figure;Fig 1.tif

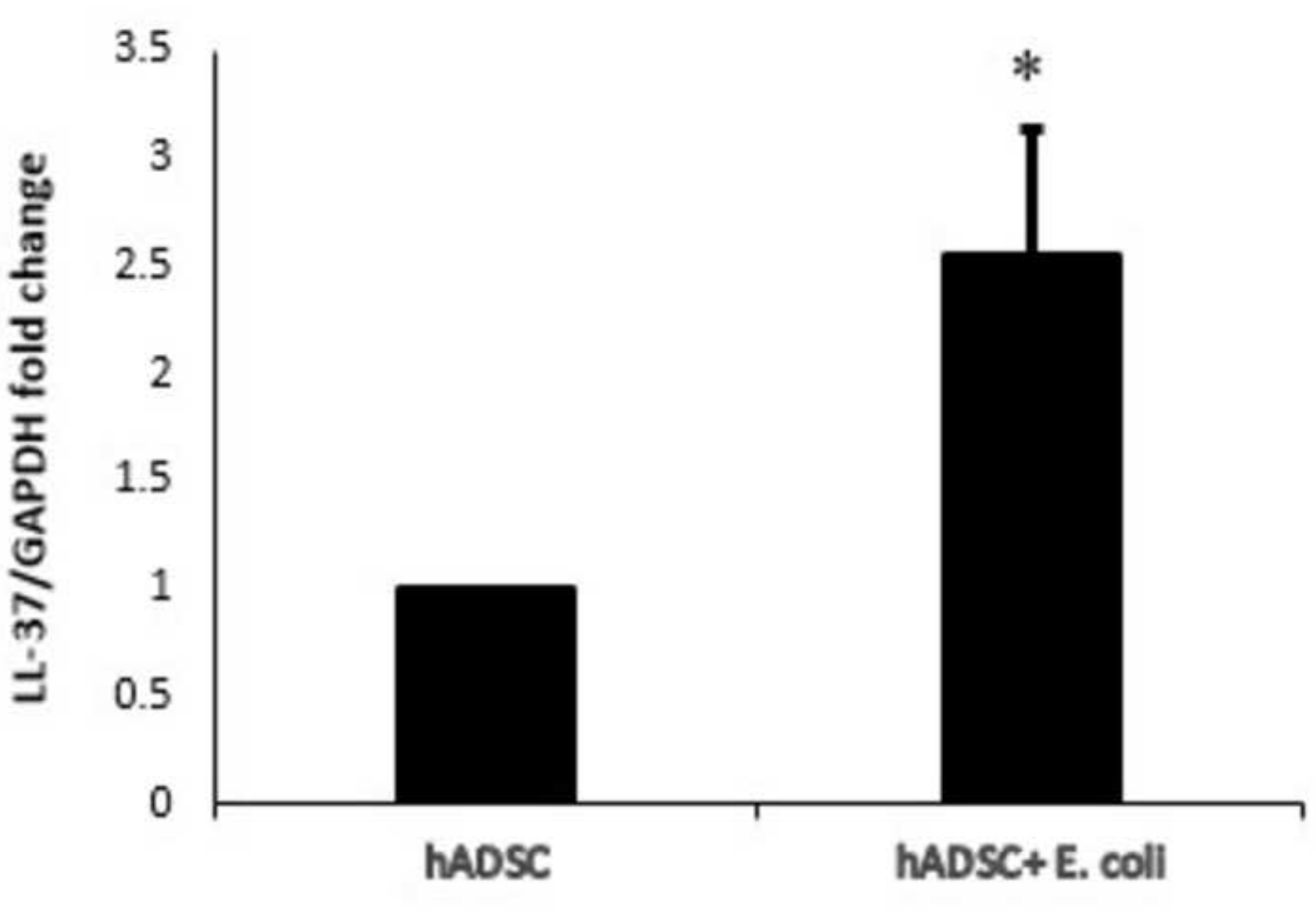

Fig 2

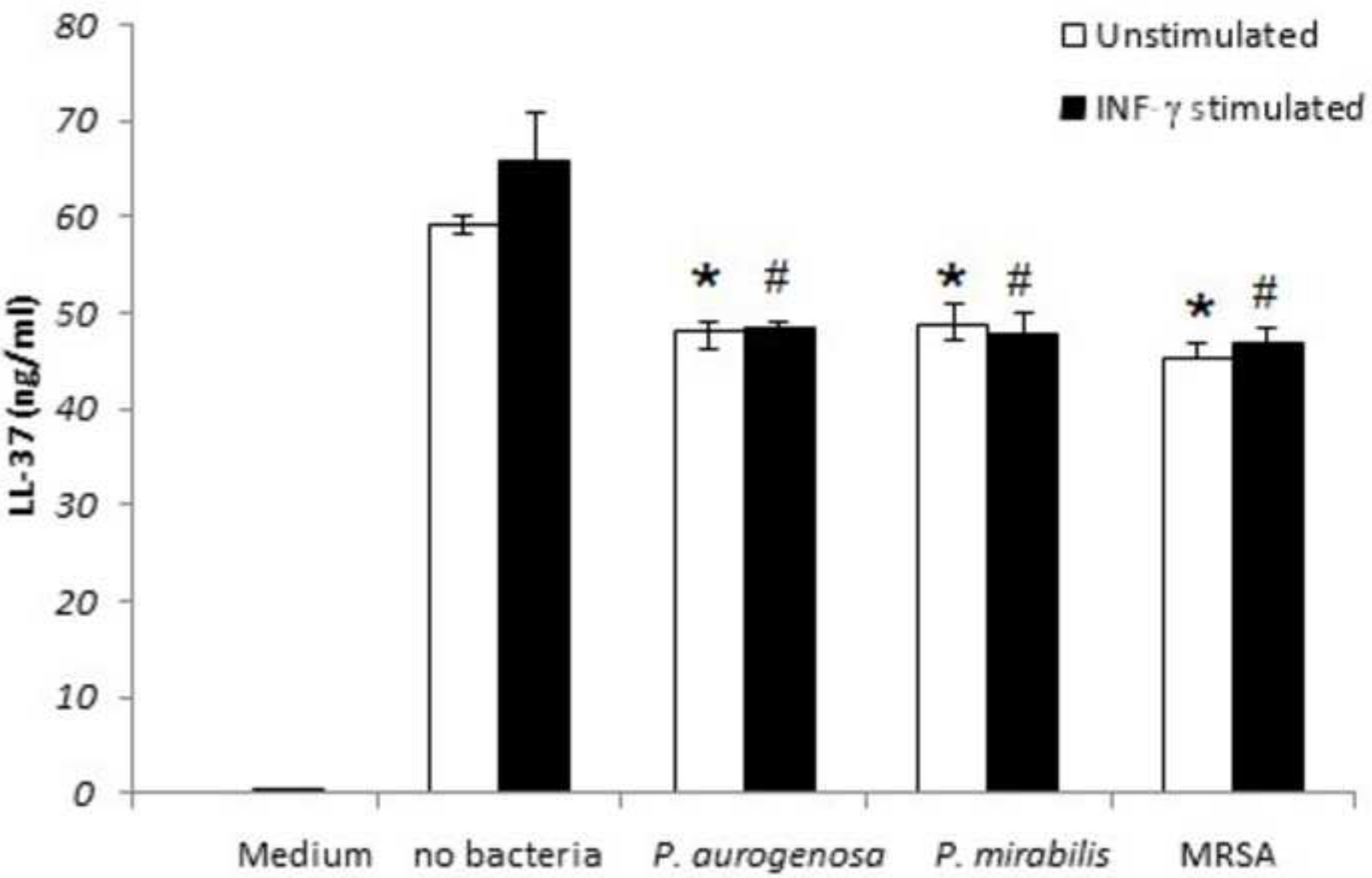
80
70
60
50
40
30
20
10
0
LL-37 (ng/ml)
Unstimulated
INF-γ stimulated
*
#
Medium
no bacteria
P. aurogenosa
P. mirabilis
MRSA

 Click here to access/download;Figure;Fig 3a.tif

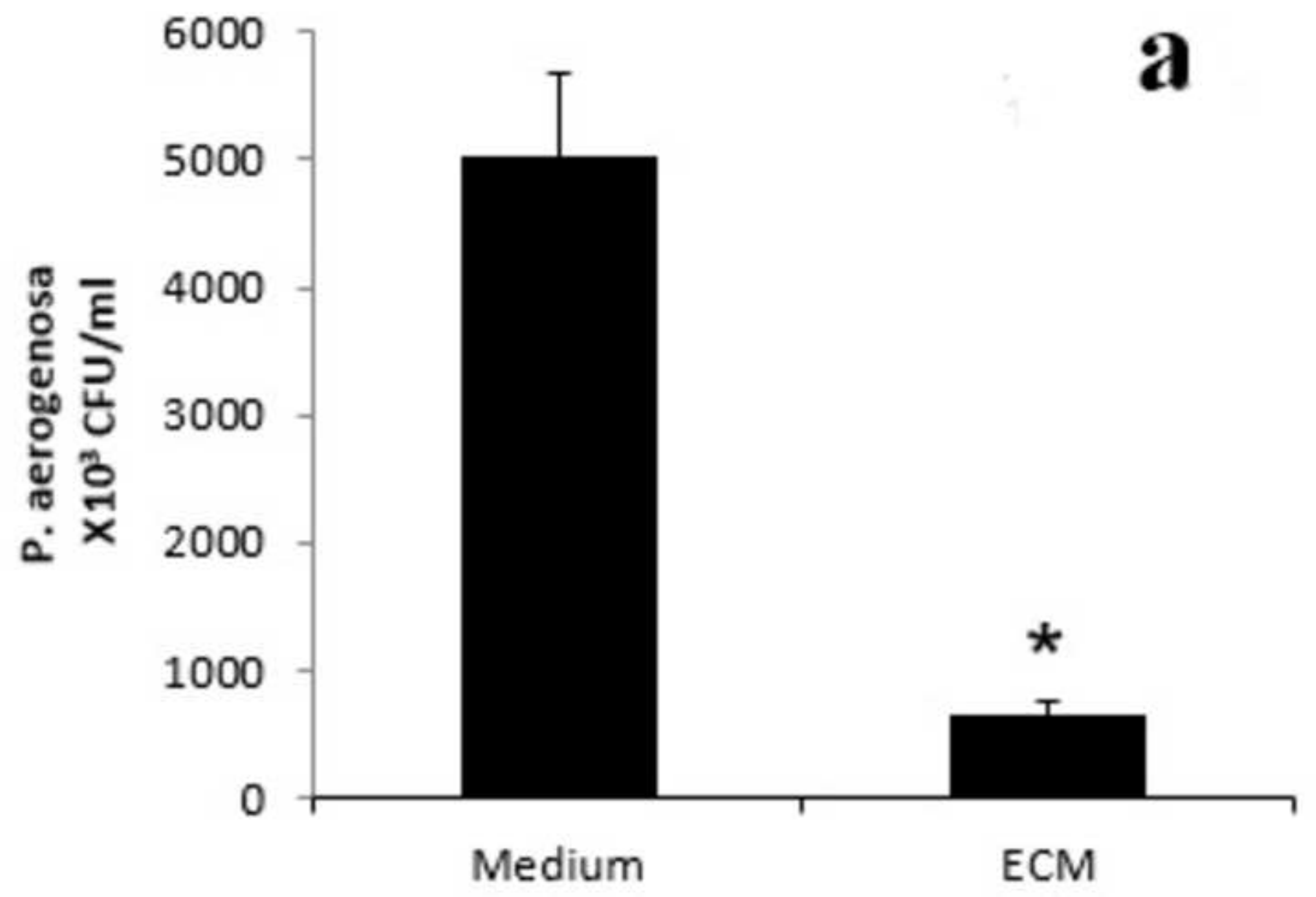

Click here to access/download;Figure;Fig 3b.tif

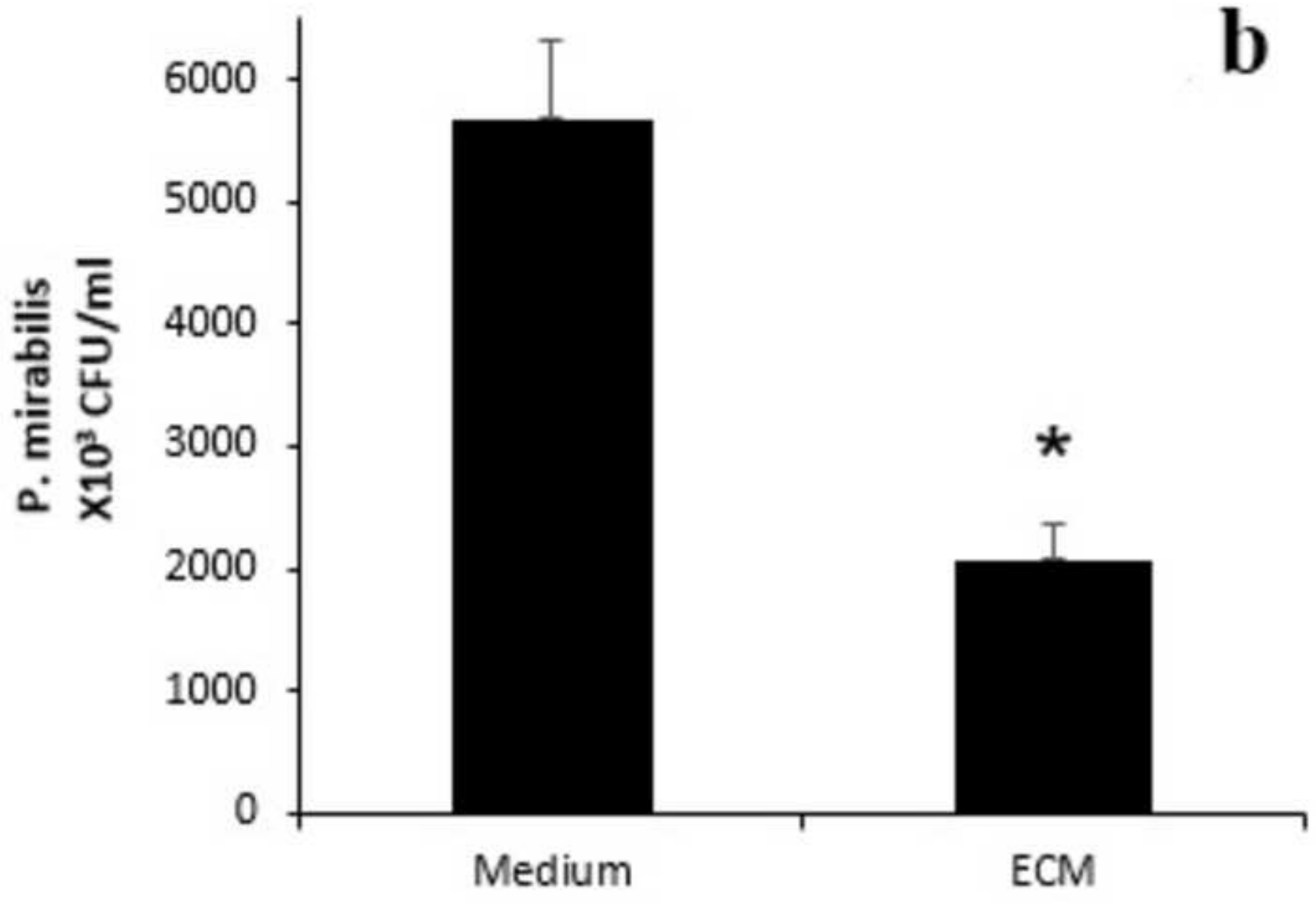

Click here to access/download;Figure;Fig 3c.tif

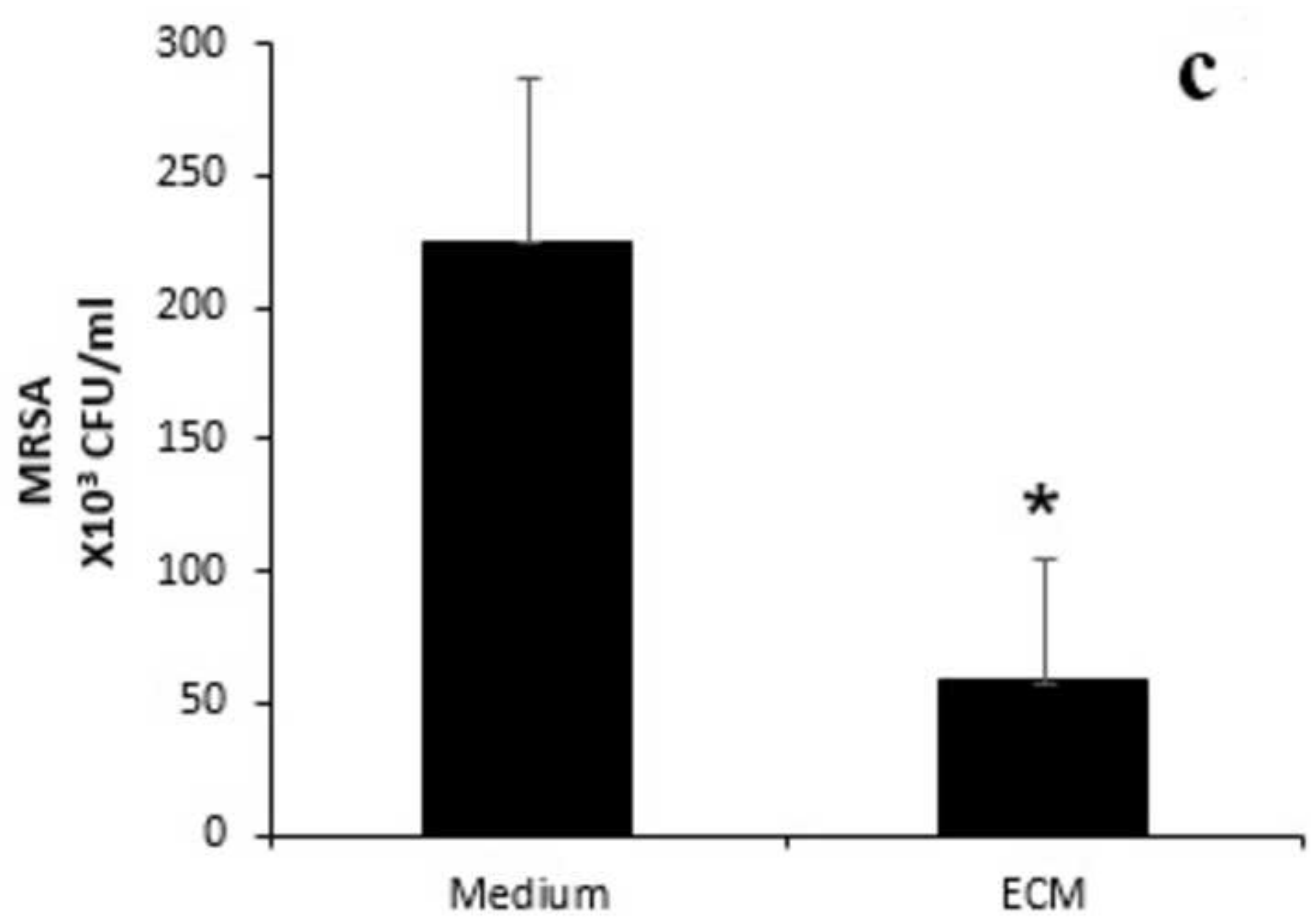

Click here to access/download;Figure;Fig 4a.tif

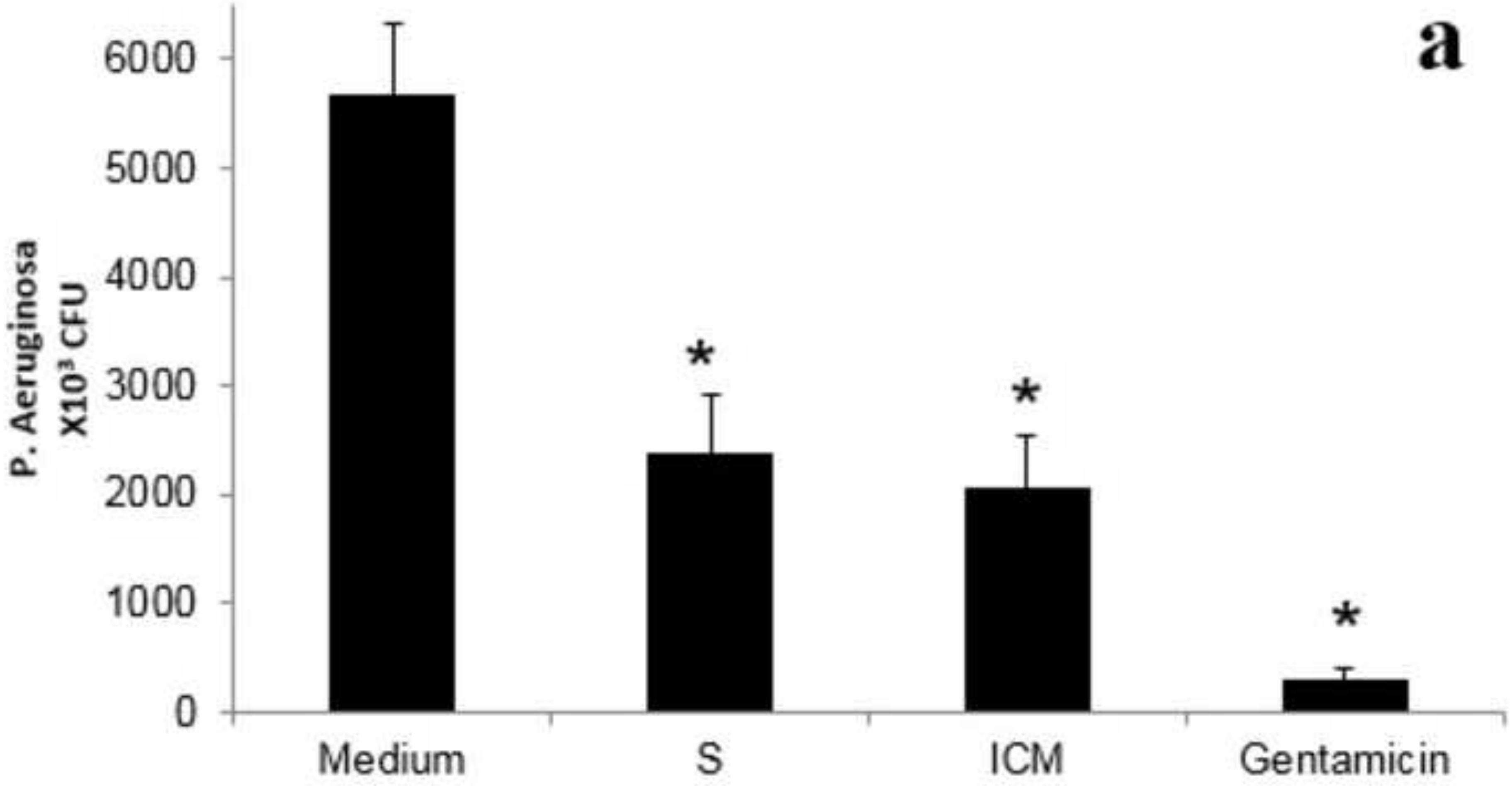

Click here to access/download;Figure;Fig 4b.tif

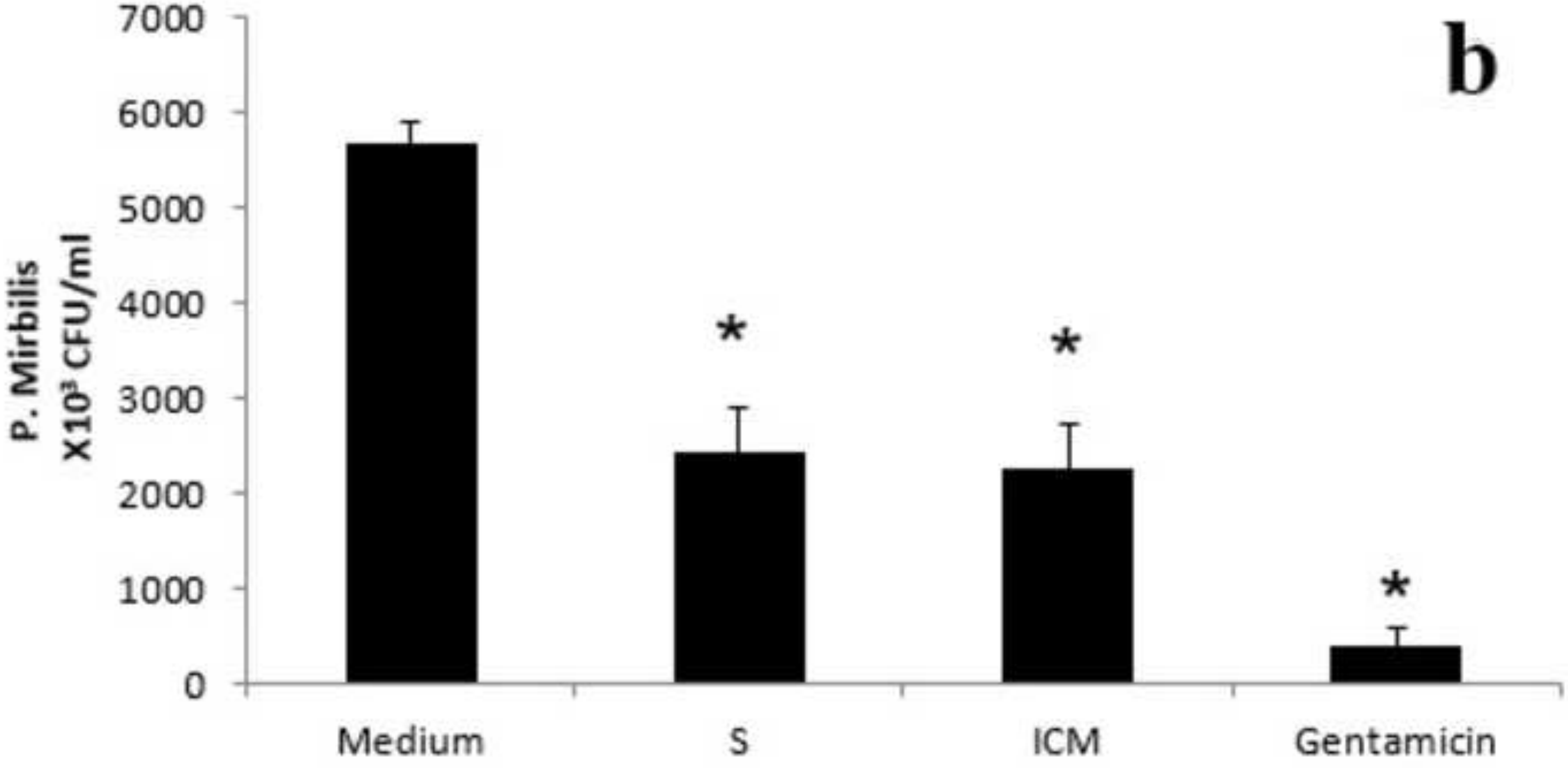

 Click here to access/download;Figure;Fig 4c.tif

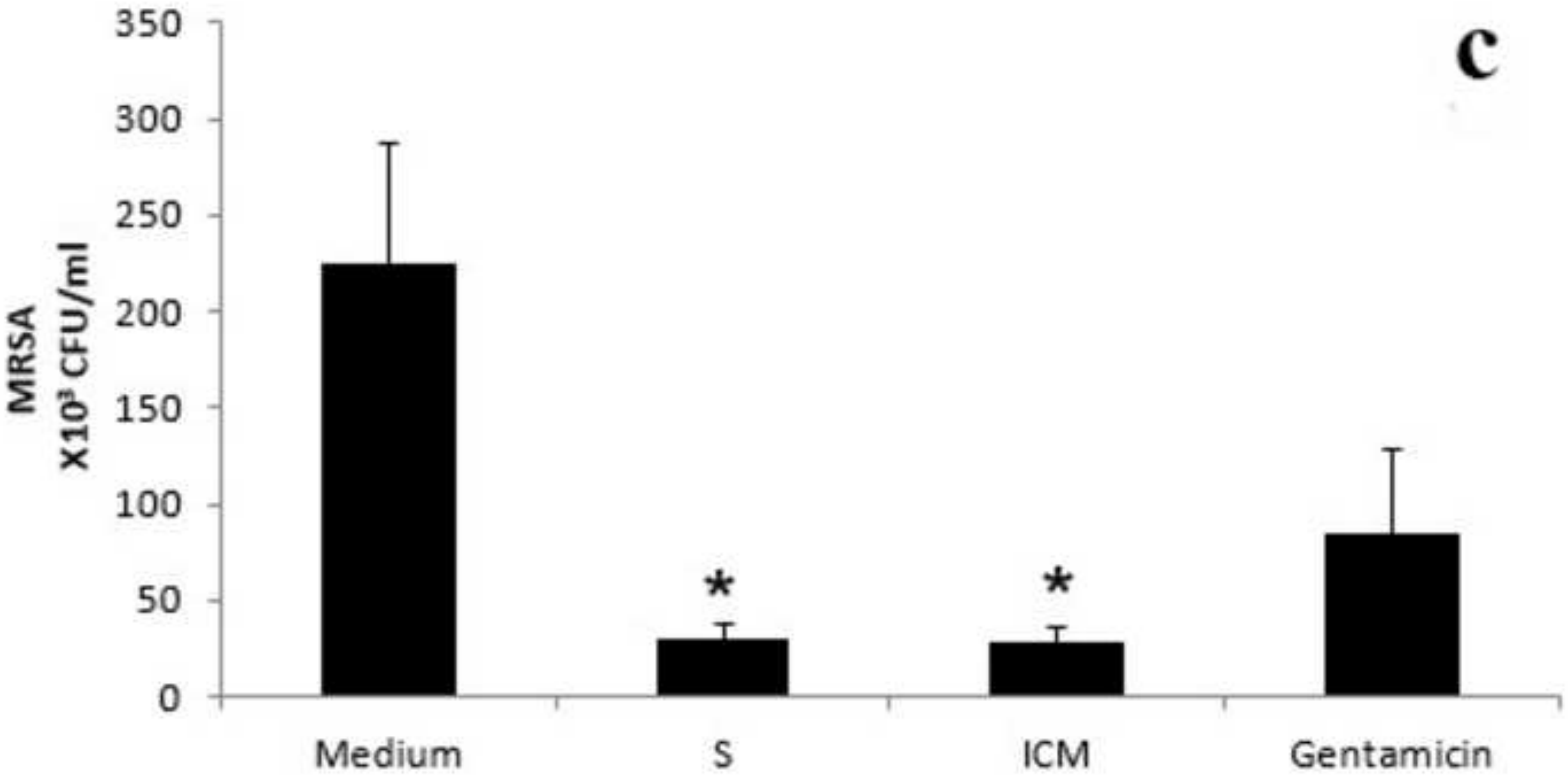

S1a_Fif

Click here to access/download
Supporting Information
S1a_ Fig..tif

S1b_Fig

Click here to access/download
Supporting Information
S1b_Fig..tif

S2_Fig

Click here to access/download
Supporting Information
S2_Fig.tif

S3a_Fig

Click here to access/download
Supporting Information
S3a_Fig..tif

S3b_Fig

Click here to access/download
Supporting Information
S3b_Fig..tif

S3c_Fig

Click here to access/download
Supporting Information
S3c_Fig..tif

S3d_Fig

Click here to access/download
Supporting Information
S3d_Fig..tif
