# Supplementary material for: The antibacterial effect of human adipose-derived stem cells on LL-37-resistant bacteria
Source: PLoS One. 2025 Oct 17;20(10):e0333647. doi: 10.1371/journal.pone.0333647 (PMC12533887; doi:10.1371/journal.pone.0333647)
Supplement: Attachment — Submitted filename: Response to Reviewers.docx [file pone.0333647.s052.docx]

Response to Editor and Reviewers’ Comments

Dear Editor and Reviewers,

We sincerely appreciate the time and effort you have dedicated to reviewing our manuscript. We have thoroughly addressed all comments and suggestions to improve the clarity, rigor, and compliance of our work with PLOS ONE guidelines. Below is our point-by-point response to each comment, along with a summary of specific changes made in the revised manuscript.

**Response to Editor’s Comments**

Manuscript Formatting and Style

We carefully reviewed and reformatted the manuscript in accordance with the official PLOS ONE style requirements for the main text, title page, authors, and affiliations. This included file naming, section headings, consistent font sizes, and reference style to fully comply with journal requirements.

Citation of Suggested Publications

We thank the reviewers for suggesting that additional references be added in specific section to support certain statements. Although no particular publications were recommended, we carefully reviewed the manuscript and inserted relevant citations where appropriate to strengthen the discussion.

Reference List Accuracy and Retraction Status

We have conducted a thorough review of our reference list and, to the best of our knowledge, none of the cited articles have been retracted. DOIs for two references were verified, corrected, and formatted according to PLOS ONE style.

Figures and PACE Tool Usage

All figures were prepared according to PLOS ONE guidelines using the PACE (PLOS Article Content Editing) tool to ensure correct resolution, dimensions, and font embedding. Figures were uploaded separately as required, improving quality and compliance.

**Response to Reviewer 1 Comments**

Use of Pronouns

We revised the manuscript to minimize use of personal pronouns such as “We” and “Our.” Formal alternatives such as “This study,” “The present study,” or “The current study” is now used consistently.

Italicization of Scientific Names

All scientific names of bacterial species have been updated and italicized throughout the manuscript, consistent with taxonomic convention (e.g*., P. aeruginosa, P. mirabilis*).

Abbreviation Table

The abbreviation table was added based on Reviewer 1’s suggestion to aid reader comprehension. While PLOS ONE guidelines do not explicitly require such a table, we included it to address the reviewer’s helpful recommendation.

**Response to Reviewer 2 Comments**

Rephrasing:

The indicated sentence was rephrased to improve clarity and flow.

Clarification of the compound:

We added the phrase “a natural human antimicrobial peptide important in the immune defense” and the sentence “Some bacteria have evolved mechanisms to evade the antimicrobial effects of LL-37” to clearly identify and explain the compound.

“Identified by” or “Differentiated by”:

We selected the term “identified by” to convey a precise and unambiguous description of the methods used.

Clarification of groups and stimulation:

We have added the sentence: “Divided into three groups: unstimulated, stimulated with interferon-gamma (IFN-γ; 100 ng/mL), or *Escherichia coli (E. coli*; 300 CFU),” and inserted “in the *E. coli*-stimulated group” in the appropriate section to clarify group distinctions.

Clarification of “IDT”:

“Integrated DNA Technologies (IDT)” has been introduced upon first mention.

Font size issue:

The font size in the Statistical analysis subsection of the Methods was increased to 12 pt for clarity and consistency with journal standards.

References missing or insufficient:

References 12, 13, and 14 were added where needed to provide appropriate support.

Italicization of species names and formatting adjustments:

Species names were corrected to italic font (e.g., *P. aeruginosa* [5 cases], *P. mirabilis* [4 cases]). The “Discussion” heading was reformatted in bold with an18-pt font size to match PLOS One style.

If any additional modifications or clarifications are needed, we will be pleased to respond promptly. We thank the Editor and Reviewers once again for their valuable feedback and hope that the revised manuscript meets the standards for publication.

Respectfully,

Dr Malek Moien Ansar

On behalf of all authors

Neuroscience Research Center, Trauma Institute, Guilan University of Medical Sciences, Guilan, Rasht, Iran

Department of Biochemistry, School of Medicine, Guilan University of Medical Sciences, Guilan, Rasht, Iran

15 August 2025
